# Bidirectional ventricular tachycardia unmasked by beta-blockade in pheochromocytoma presenting as hypertensive optic neuropathy: a case report and literature review

**DOI:** 10.3389/fmed.2026.1942798

**Published:** 2026-10-08

**Authors:** Jianhua Xie, Qin Yuxue, Hai Huang, Xiaojia Luo

**Affiliations:** Department of Cardiovascular Medicine, West China School of Medicine, Sichuan University; Sichuan University Affiliated Chengdu Second People’s Hospital, Chengdu Second People’s Hospital, Chengdu, Sichuan, China

**Keywords:** beta-blocker, bidirectional ventricular tachycardia, case report, catecholamine, pheochromocytoma, secondary hypertension

## Abstract

**Background:**

Pheochromocytoma is a catecholamine-secreting neuroendocrine tumor that can produce life-threatening cardiovascular complications. Bidirectional ventricular tachycardia (BVT) is a rare arrhythmia with a narrow differential diagnosis, classically associated with digitalis toxicity and catecholaminergic polymorphic ventricular tachycardia, and only exceptionally reported as the presenting feature of pheochromocytoma.

**Case presentation:**

A 51-year-old woman presented with acute visual impairment secondary to hypertensive optic neuropathy; her blood pressure was 220/138 mmHg. Within 24 h of initiating sodium nitroprusside and metoprolol, she developed bidirectional ventricular tachycardia followed by torsades de pointes. Marked blood-pressure lability, dramatically elevated plasma free metanephrines and catecholamines, and a left adrenal mass on computed tomography established the diagnosis of pheochromocytoma. After alpha-adrenergic blockade with phenoxybenzamine, laparoscopic adrenalectomy was performed, and histopathology confirmed the diagnosis. No ventricular arrhythmia recurred, and blood pressure normalized without antihypertensive medication.

**Conclusion:**

Bidirectional ventricular tachycardia may be the initial manifestation of pheochromocytoma and may be unmasked by beta-blockade administered before alpha-blockade. Unexplained BVT accompanied by blood-pressure fluctuations should prompt biochemical screening and adrenal imaging. Etiologic treatment is curative, whereas antiarrhythmic therapy alone is insufficient. We review the previously published cases to contextualize this rare phenotype.

## Introduction

Pheochromocytomas and paragangliomas (PPGLs) are neuroendocrine tumors arising from chromaffin cells of the adrenal medulla and extra-adrenal paraganglia, respectively (1). Their combined annual incidence is approximately 1–8 per million population (2, 3), and up to one-quarter are discovered incidentally (4). The 2022 World Health Organization classification regards all PPGLs as having metastatic potential (5).

Cardiovascular manifestations dominate the clinical morbidity of these tumors. Catecholamine excess drives cyclic AMP-mediated calcium influx and spontaneous sarcoplasmic reticulum calcium release, producing intracellular calcium overload that lowers the threshold for delayed afterdepolarizations and triggered ventricular activity (6). Patients may develop cardiomyopathy, myocarditis, acute coronary syndromes, and malignant arrhythmias (6). Diagnosis rests on biochemical evidence of catecholamine overproduction—plasma free or urinary fractionated metanephrines being the recommended initial test—followed by cross-sectional imaging and germline genetic testing (4).

Bidirectional ventricular tachycardia (BVT) is a rare ventricular arrhythmia defined by beat-to-beat alternation of the QRS axis, first described in 1922 in digitalis toxicity (7). It has subsequently been reported in catecholaminergic polymorphic ventricular tachycardia (CPVT), Andersen-Tawil syndrome, myocarditis, cardiac sarcoidosis, hypokalemia, and stress-induced cardiomyopathy (8, 9). Mechanistically, BVT reflects triggered activity from intracellular calcium overload; in selected cases, a ping-pong mechanism within the His-Purkinje system produces alternating left- and right-axis deviation (10). BVT is clinically ominous because of its propensity to degenerate into polymorphic ventricular tachycardia or ventricular fibrillation (8, 10).

Pheochromocytoma presenting with BVT is exceptionally rare. Herein we report such a case, distinguished by hypertensive optic neuropathy as the heralding symptom and ventricular arrhythmia temporally unmasked by beta-blockade administered before alpha-blockade. We contextualize the case against all previously published reports. This report was prepared in accordance with the CARE guidelines (12).

## Case presentation

A 51-year-old woman was admitted in June 2025 with a 3-day history of progressive visual impairment. She had a 10-year history of anxiety and depression managed with paroxetine and olanzapine. She denied hypertension, diabetes, coronary artery disease, or substance use.

On admission, she appeared anxious and markedly hypertensive (220/138 mmHg). Physical examination revealed tachycardia (117 beats/min), regular rhythm, and no cardiopulmonary or abdominal abnormalities. Initial electrocardiography demonstrated sinus tachycardia, left ventricular high voltage, ST-T changes, and QTc prolongation to 496 ms (Figure 1A). Ophthalmologic consultation confirmed bilateral acute hypertensive optic neuropathy. Given severely elevated blood pressure and tachycardia, intravenous sodium nitroprusside and oral metoprolol were initiated.

**Figure 1:**
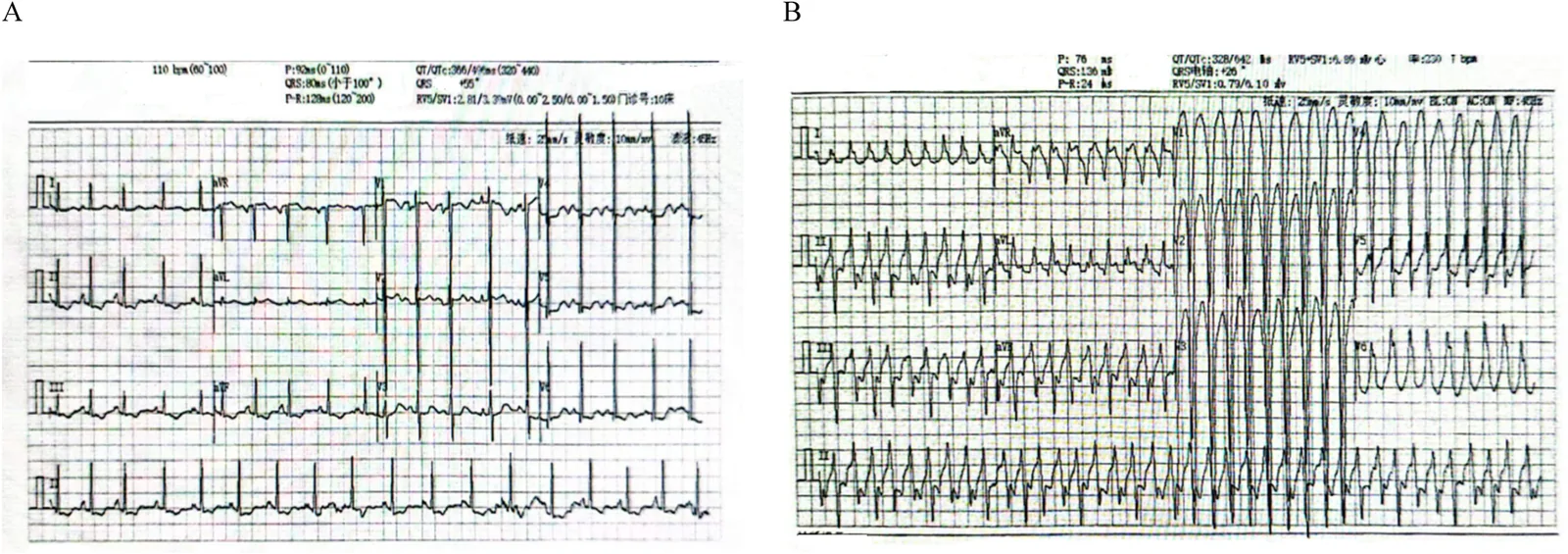
**(A)** electrocardiogram on admission showing sinus tachycardia, normal axis, left ventricular high voltage, ST-T changes, and QTc prolongation (496 ms). **(B)** Electrocardiogram during ventricular tachycardia episode demonstrating bidirectional QRS axis alternation.

On the following day, she developed sustained ventricular tachycardia with beat-to-beat QRS axis alternation, consistent with BVT (Figure 1B). Intravenous esmolol was administered. Emergent transthoracic echocardiography revealed left atrial enlargement, mild regional wall-motion abnormalities, mild valvular regurgitation, trace pericardial effusion, and preserved left ventricular ejection fraction of 58%. Continuous monitoring captured episodes of torsades de pointes (Figure 2). Serum electrolytes remained stable throughout: potassium 3.9–4.3 mmol/L (reference 3.5–5.0) and magnesium 0.82–0.91 mmol/L (reference 0.70–1.10), measured on admission and repeated daily during the arrhythmic episodes. There was no evidence of hemoconcentration (hemoglobin 142 g/L, hematocrit 41%, albumin 38 g/L) or tumor lysis (LDH 198 U/L, creatinine 78 μmol/L, BUN 6.2 mmol/L, alkaline phosphatase 76 U/L).

**Figure 2:**
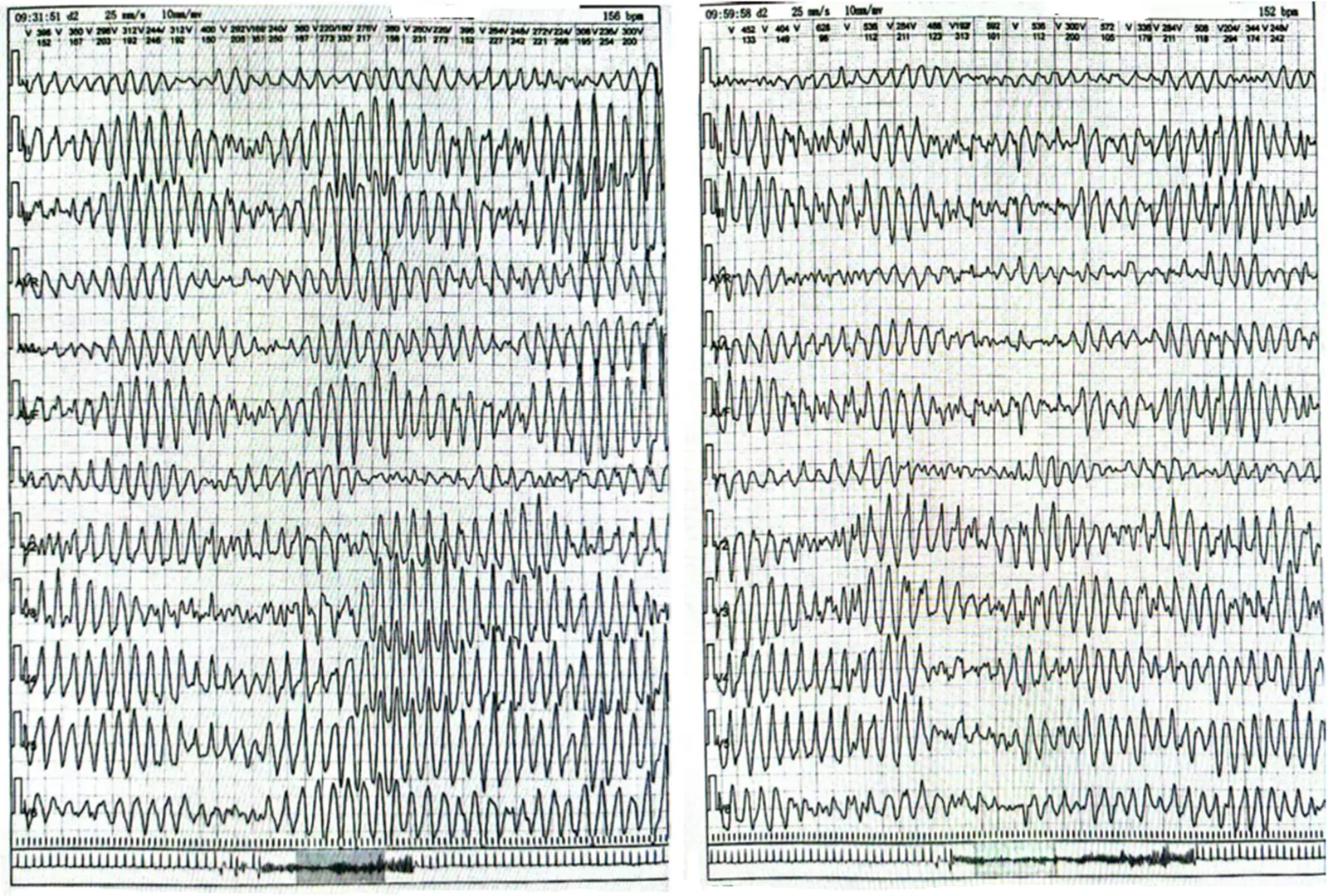
Telemetry strip demonstrating episodes of torsades de pointes during continuous cardiac monitoring.

The arrhythmias were initially managed as a primary cardiac disorder; however, ventricular ectopy recurred despite escalating antiarrhythmic therapy, prompting a systematic search for underlying causes. A temporary transvenous pacemaker was implanted for overdrive suppression. Retrospective review of blood-pressure recordings revealed dramatic fluctuations (Figure 3), raising suspicion for pheochromocytoma. Other secondary causes of hypertension were evaluated and excluded: plasma aldosterone/renin ratio was normal, renal artery Doppler ultrasound and renal function were unremarkable, and there were no clinical or biochemical features of Cushing syndrome.

**Figure 3:**
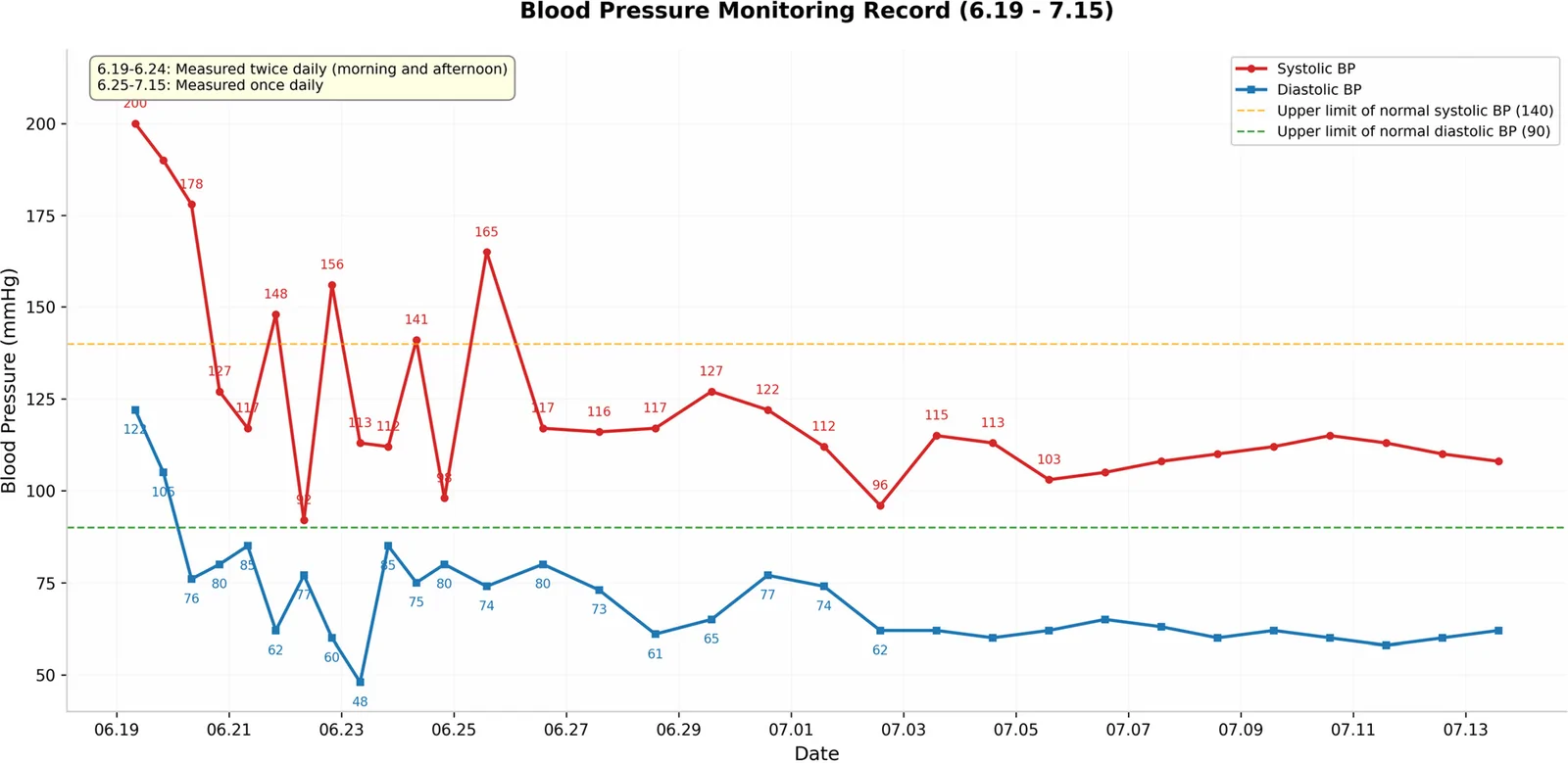
Trend of systolic and diastolic blood pressure recordings throughout the hospital course, demonstrating marked fluctuations characteristic of pheochromocytoma.

Biochemical evaluation demonstrated markedly elevated plasma free metanephrines: metanephrine 8.11 nmol/L (reference <0.5) and normetanephrine 15.93 nmol/L (reference <0.9), together with dopamine 137.7 pmol/L, epinephrine 14,157.8 pmol/L, and norepinephrine 19,964.7 pmol/L. Twenty-four-hour urinary vanillylmandelic acid excretion was 22.08 mg/24 h. Adrenal computed tomography confirmed a large left adrenal mass (Figure 4).

**Figure 4:**
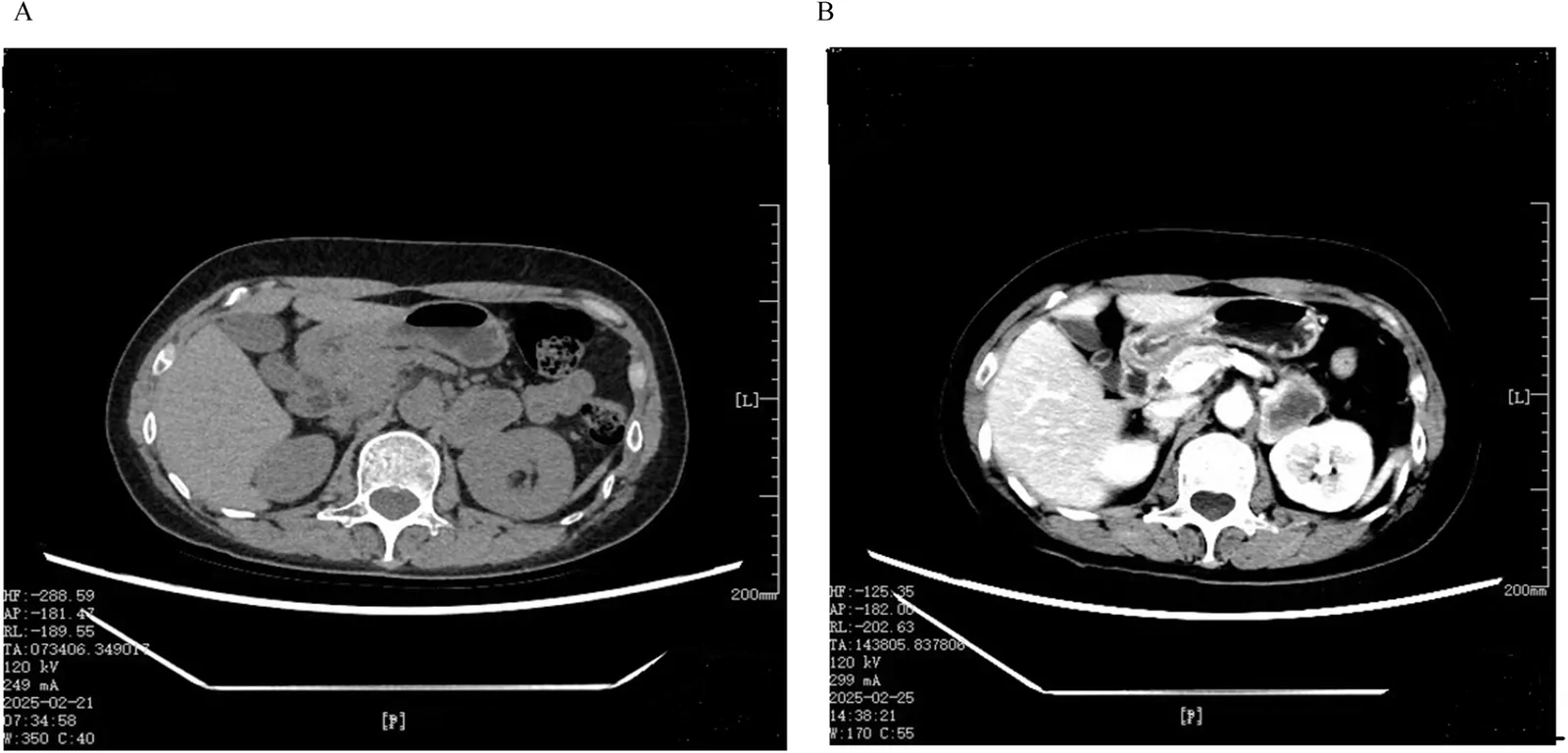
**(A)** adrenal computed tomography (axial view) demonstrating a large left adrenal mass. **(B)** Adrenal computed tomography (coronal view) demonstrating a large left adrenal mass.

Preoperative alpha-adrenergic blockade was established with phenoxybenzamine, initiated at 10 mg twice daily and titrated over 10 days to 30 mg three times daily. Concurrent intravenous crystalloid infusion at 120 mL/h and liberal oral salt intake were administered for volume expansion. After adequate alpha-blockade was achieved, oral metoprolol 25 mg twice daily was added for reflex tachycardia. After 2 weeks of preparation, the patient underwent laparoscopic left adrenalectomy. Intraoperatively, she remained hemodynamically stable with only transient blood pressure elevation (maximum 165/95 mmHg during tumor manipulation) managed with incremental nicardipine; no vasopressors were required postoperatively. She was extubated uneventfully and monitored in the intensive care unit for 24 h.

Gross examination revealed a well-circumscribed, tan-brown adrenal mass measuring 2.5 × 2.5 × 1.8 cm. Histopathology demonstrated classic Zellballen architecture, with nests of polygonal chief cells separated by a rich capillary network (Figure 5A). Tumor cells exhibited abundant granular eosinophilic cytoplasm and round-to-oval nuclei with salt-and-pepper chromatin (Figure 5B). Immunohistochemistry was strongly positive for chromogranin A, synaptophysin, and S100 protein (sustentacular cells), and negative for pan-cytokeratin, HMB45, Melan A/MART-1, inhibin-alpha, and PAX-8; the Ki-67 index was approximately 2%.

**Figure 5:**
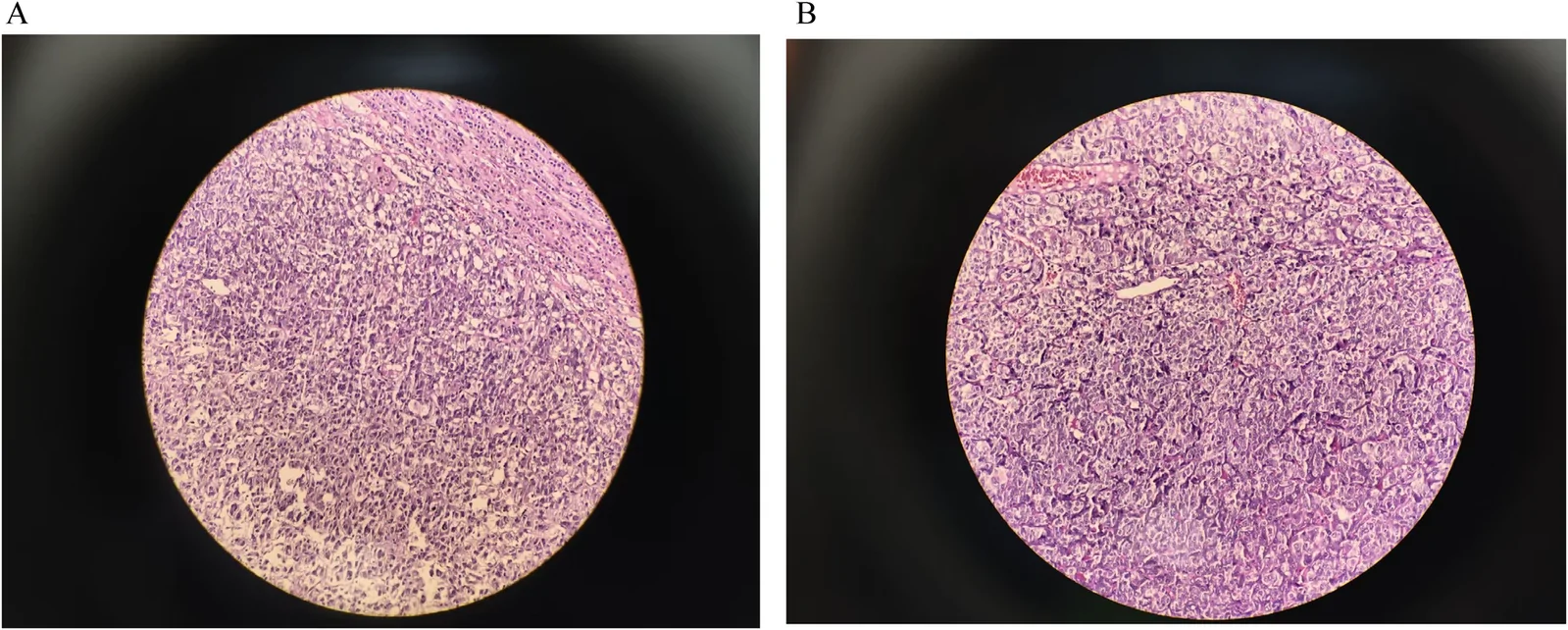
**(A)** histopathological examination of the resected adrenal mass (H&E staining) demonstrating the classic “Zellballen” architecture, characterized by nests of polygonal neuroendocrine cells separated by a rich vascular stroma (original magnification ×100). **(B)** Higher magnification view (H&E staining) showing tumor cells with abundant granular eosinophilic cytoplasm and round to oval nuclei with characteristic “salt-and-pepper” chromatin (original magnification ×200).

Following resection, no further ventricular tachycardia occurred, and the temporary pacemaker was removed. Blood pressure normalized without antihypertensive medication, and she was discharged in stable condition. At outpatient follow-up, she remains normotensive and free of arrhythmia. Her acute catecholamine-related anxiety and palpitations resolved; however, her underlying generalized anxiety disorder and depression, present for 10 years, persisted at a milder intensity, and she continued outpatient psychiatric care with paroxetine and olanzapine. The clinical course is summarized in Table 1.

**Table 1:** Timeline of the clinical course, diagnostic evaluation, and management.

**Time**	**Clinical event**	**Key findings**	**Management**
Day 1 (admission)	Acute progressive visual impairment; blood pressure 220/138 mmHg	Bilateral hypertensive optic neuropathy; ECG: sinus tachycardia, left ventricular high voltage, ST-T changes, QTc 496 ms (Figure 1A)	Intravenous sodium nitroprusside; oral metoprolol
Day 2	Onset of sustained ventricular tachycardia	ECG: bidirectional ventricular tachycardia (Figure 1B); echocardiography: left atrial enlargement, mild regional wall-motion abnormality, LVEF 58%	Intravenous esmolol; continuous cardiac monitoring
Days 3–5	Recurrent arrhythmia	Holter: torsades de pointes (Figure 2); marked blood-pressure fluctuations (Figure 3)	Temporary transvenous pacemaker for overdrive suppression; antihypertensive therapy changed to terazosin; workup for secondary hypertension initiated
Weeks 1–2	Etiological work-up	Plasma metanephrine 8.11 nmol/L and normetanephrine 15.93 nmol/L; epinephrine 14,157.8 and norepinephrine 19,964.7 pmol/L; CT: left adrenal mass (Figure 4)	Diagnosis of pheochromocytoma; phenoxybenzamine 10 mg BID titrated to 30 mg TID over 10 days; intravenous crystalloid 120 mL/h; oral salt loading
Week 3	Laparoscopic left adrenalectomy	Tumor 2.5 × 2.5 × 1.8 cm; Zellballen architecture; chromogranin A, synaptophysin and S100 positive; Ki-67 ∼2% (Figure 5)	Surgical resection after adequate alpha-blockade; intraoperative hemodynamics stable; 24-h ICU monitoring
Postoperative follow-up	Uneventful recovery	No further ventricular arrhythmia; blood pressure normal without medication; pacemaker removed; anxiety improved but underlying psychiatric condition persisted	Discharge; outpatient surveillance with periodic metanephrine measurement; continued psychiatric follow-up

## Discussion

This case illustrates three clinically important points. First, pheochromocytoma may declare itself through malignant ventricular arrhythmia arising in the setting of hypertensive target-organ damage rather than the classic triad. Second, the arrhythmia emerged shortly after beta-blockade without preceding alpha-blockade, an association with direct implications for initial management. Third, once the tumor was resected after appropriate alpha-adrenergic preparation, the arrhythmia was abolished and blood pressure normalized, underscoring that pheochromocytoma is a curable cause of BVT.

## Mechanisms of bidirectional ventricular tachycardia in pheochromocytoma

The pathophysiological link between pheochromocytoma and BVT is incompletely understood, but several mechanisms likely contribute. Direct catecholamine cardiotoxicity induces cyclic AMP-mediated calcium influx and spontaneous release, leading to intracellular calcium overload that triggers delayed afterdepolarizations (6). Catecholamines additionally provoke coronary vasospasm and increase left ventricular afterload, compounding supply-demand mismatch (6). Our patient's plasma catecholamine concentrations, exceeding upper limits by more than an order of magnitude, support this mechanism.

A second proposed mechanism involves the His-Purkinje system. In some BVT cases, the QRS duration is relatively narrow, suggesting participation of the specialized conduction system. Baher et al. proposed a ping-pong mechanism in which alternating exit sites generate alternating left- and right-axis deviation; the alternation may be sustained by reciprocal activation of two ventricular foci with different triggering thresholds (10). This framework accounts for the regular beat-to-beat QRS alternation observed in our patient.

Third, repolarization abnormalities may act synergistically. Our patient exhibited baseline QTc prolongation (496 ms) and subsequently developed torsades de pointes. Catecholamines can prolong ventricular repolarization, and concomitant paroxetine and olanzapine therapy, although only weakly associated with QT prolongation, may have contributed to reduced repolarization reserve. Serum potassium and magnesium remained within normal limits, excluding electrolyte depletion as a cofactor (13).

Fourth, chronic catecholamine exposure produces a structural substrate. Cardiovascular magnetic resonance studies have demonstrated catecholamine-mediated myocarditis, focal and diffuse fibrosis, and myocardial dysfunction (14); these changes are at least partially reversible once catecholamine excess is abolished. In our patient, however, left ventricular ejection fraction was preserved and wall-motion abnormalities were mild, suggesting a predominantly functional, catecholamine-driven substrate rather than established structural heart disease—a distinction that likely explains complete arrhythmia resolution after tumor resection.

## Atypical presentations of pheochromocytoma

Although the classic triad of headache, palpitations, and diaphoresis is well recognized, pheochromocytoma frequently presents with atypical or organ-specific manifestations that delay diagnosis. Recent reports have highlighted incidental adrenal masses discovered on unrelated imaging, treatment-resistant hypertension, acute heart failure, chest pain, gastrointestinal symptoms (notably refractory vomiting), and cerebrovascular events as heralding features (21–25). In a single-center series of 94 patients, Falhammar et al. found that although hypertension was common, the presenting symptoms were highly heterogeneous and often mimicked primary cardiac, gastrointestinal, or psychiatric disorders (25). The biochemical phenotype (adrenergic vs. noradrenergic) partially explains this clinical diversity. Our patient's presentation with acute visual loss from hypertensive optic neuropathy, rather than classic paroxysmal symptoms, exemplifies this diagnostic challenge. Clinicians should maintain a low threshold for biochemical screening when hypertension is severe, labile, or accompanied by unusual target-organ injury.

## Comparison with previously published cases

We reviewed the English-language literature and identified four previously reported cases of pheochromocytoma presenting with BVT (Table 2). Traykov et al. described a 44-year-old woman with recurrent hypertensive crises in whom BVT was converted to sinus rhythm before the underlying tumor was recognized (15). Quina-Rodrigues et al. reported a 70-year-old man with an ACTH-producing pheochromocytoma who presented with severe metabolic alkalosis and profound hypokalemia (1.48 mmol/L); BVT was attributed to synergistic calcium overload combined with severe hypokalemia (16). Li et al. described a 55-year-old man whose BVT was initially misdiagnosed as acute viral myocarditis; glucocorticoid administration precipitated a pheochromocytoma crisis (17). Lopez-Pena et al. reported a 27-year-old woman who presented with BVT and cardiogenic shock caused by acute hemorrhagic necrosis of a large adrenal pheochromocytoma; she recovered fully after alpha-beta blockade and adrenalectomy (18).

**Table 2:** Published cases of pheochromocytoma presenting with bidirectional ventricular tachycardia.

**Study (year, journal)**	**Age/sex**	**Initial presentation**	**Precipitating context**	**Key cardiac features**	**Management and outcome**
Traykov et al. Heart (15)	44/F	Recurrent hypertensive crises	Hypertensive episode	BVT with frontal-plane QRS axis alternans	Converted to sinus rhythm; pheochromocytoma recognized on subsequent work-up
Quina-Rodrigues et al., Europace (16)	70/M	BVT requiring cardioversion	ACTH-producing tumor; severe hypokalemia (1.48 mmol/L) and metabolic alkalosis	BVT; two direct-current cardioversions	Cardioversion and potassium repletion; synergistic catecholamine-hypokalemia mechanism proposed
Li et al. Ann Noninvasive Electrocardiol (17)	55/M	BVT as first manifestation; initially misdiagnosed as viral myocarditis	Glucocorticoid administration precipitated hypertensive crisis	BVT; acute heart failure with reduced ejection fraction	Alpha-blockade followed by adrenalectomy; arrhythmia resolved
Lopez-Pena et al. Eur Heart J Case Rep (18)	27/F	Cardiogenic shock	Acute hemorrhagic necrosis of a 7.1 × 6.6 cm adrenal pheochromocytoma	BVT; dilated left ventricle with severe systolic dysfunction	Alpha-beta blockade then laparoscopic adrenalectomy; full recovery of ejection fraction; genetic testing negative
Present case	51/F	Acute visual loss from hypertensive optic neuropathy	BVT and torsades de pointes emerged after beta-blockade without prior alpha-blockade	BVT degenerating to torsades; QTc 496 ms; preserved LVEF 58%	Phenoxybenzamine then laparoscopic adrenalectomy; no arrhythmia recurrence; normotensive without medication

Our case differs from these reports in several respects. The heralding manifestation was acute visual loss from hypertensive optic neuropathy rather than palpitations, syncope, heart failure, or shock. Ventricular arrhythmia emerged in close temporal relation to beta-blockade initiation, before pheochromocytoma was suspected and before alpha-blockade. Left ventricular systolic function was preserved, and no electrolyte disturbance, glucocorticoid exposure, or digitalis use was present.

Viewed collectively, these cases reveal recurring patterns. A triggering event is frequently identifiable: glucocorticoid administration (17), beta-blockade without preceding alpha-blockade (present case), severe electrolyte depletion (16), or acute tumor hemorrhage (18). Arrhythmia may be the first manifestation of the tumor, and conventional antiarrhythmic therapy tends to fail until catecholamine excess is controlled. Most importantly, the prognosis after etiologic treatment is favorable: in every reported case with documented follow-up, ventricular arrhythmia resolved after adrenalectomy, and cardiac function recovered when impaired (17, 18). Bidirectional ventricular tachycardia in the appropriate clinical context should therefore be regarded as a potentially curable arrhythmia.

## Why the diagnosis was initially missed

Several features conspired to delay the diagnosis. The patient presented to a cardiology service with visual loss as the dominant complaint, and severe hypertension was attributed to a hypertensive emergency complicating essential hypertension. Her 10-year history of anxiety and depression provided a plausible alternative explanation for episodic distress, an important pitfall because paroxysmal symptoms of catecholamine excess overlap considerably with anxiety and panic attacks. In retrospect, the severity of hypertension at her age, the presence of hypertensive target-organ damage, and the subsequently documented dramatic blood-pressure lability were clues to a secondary etiology. This experience mirrors that of Li et al., whose patient was treated for presumed viral myocarditis until a glucocorticoid-provoked crisis exposed the underlying tumor (17). Both cases illustrate that pheochromocytoma is less often missed for want of sophisticated testing than for want of clinical suspicion.

## The beta-blockade lesson

The temporal relationship between beta-blocker administration and the onset of BVT deserves emphasis. In unrecognized pheochromocytoma, blockade of beta-adrenergic receptors without preceding alpha-blockade leaves alpha-adrenergic vasoconstriction unopposed, exacerbating hypertension and myocardial catecholamine toxicity; current guidelines mandate alpha-adrenergic blockade before any beta-blockade (4). The Endocrine Society guideline explicitly recommends that beta-blockade be introduced only after adequate alpha-blockade has been achieved, and then principally to control reflex tachycardia (4). Our patient received metoprolol and subsequently esmolol for sinus tachycardia and presumed essential hypertension, and BVT was documented within 24 h. Although a causal relationship cannot be proven from a single observation, the sequence is concordant with the mechanistic concern and mirrors the precipitation of crisis by glucocorticoids in the case reported by Li et al. (17). It is noteworthy that beta-blockade is not invariably deleterious in this setting: pheochromocytoma-associated ventricular tachycardia responsive to atenolol has been reported, but only in the context of established diagnosis and appropriate alpha-adrenergic control (19). For clinicians managing severe hypertension with tachycardia of unknown cause, our case argues for considering pheochromocytoma before reaching for a beta-blocker.

## Diagnostic and management implications

Because the differential diagnosis of BVT is narrow, its occurrence should trigger a structured etiological search. When BVT is accompanied by paroxysmal or labile hypertension, headache, sweating, or palpitations, measurement of plasma free or urinary fractionated metanephrines should be obtained early, followed by cross-sectional adrenal imaging when biochemistry is positive (4). Equally important is the therapeutic hierarchy: antiarrhythmic drugs and device therapy address the consequence but not the cause. Contemporary guidelines for the management of ventricular arrhythmias emphasize identification and treatment of reversible underlying etiologies (20), and pheochromocytoma is among the few genuinely curable causes of malignant ventricular arrhythmia. In our patient, antiarrhythmic therapy and temporary pacing provided only transient control, whereas alpha-blockade followed by adrenalectomy abolished the arrhythmia permanently.

In our patient, the principal alternative causes of BVT were systematically excluded. There was no digitalis exposure; serum potassium remained normal; her age, the absence of exercise-induced arrhythmia, and the lack of family history argued against CPVT (11), notwithstanding the phenotypic overlap; and echocardiography showed no structural substrate to suggest myocarditis, sarcoidosis, or cardiomyopathy (8, 9). The coexistence of severe, labile hypertension ultimately directed attention to a catecholamine-secreting tumor, illustrating how the accompanying clinical context guides the etiological search.

## Strengths and limitations

This report has limitations inherent to a single-case design. The temporal association between beta-blockade and arrhythmia onset cannot establish causality. Germline genetic testing, which current guidelines recommend for all patients with PPGLs (4), was not performed, and formal PASS or GAPP grading of the tumor was not undertaken; both are acknowledged as limitations. Follow-up to date is of limited duration, and long-term biochemical surveillance with periodic metanephrine measurement is planned in accordance with guideline recommendations (4, 5). Strengths include complete biochemical, electrocardiographic, imaging, and histopathological documentation, systematic comparison with all previously published cases, and detailed perioperative data.

## Conclusion

Bidirectional ventricular tachycardia can be the initial clinical manifestation of pheochromocytoma and may be unmasked by beta-blockade administered before alpha-blockade. The arrhythmogenic mechanism is driven by catecholamine-induced intracellular calcium overload and triggered activity, electrophysiologically akin to catecholaminergic polymorphic ventricular tachycardia. Our synthesis of published cases indicates that this phenotype, although rare, is associated with favorable outcomes once the tumor is resected, provided the diagnosis is established before irreversible cardiac injury occurs. Clinicians encountering unexplained BVT, particularly when accompanied by blood-pressure fluctuations, headache, sweating, or palpitations, should pursue early biochemical screening and adrenal imaging and should avoid beta-blockers and glucocorticoids until pheochromocytoma has been excluded or adequately alpha-blocked. Definitive surgical resection after appropriate preparation is curative and substantially improves prognosis, reinforcing the fundamental principle of treating the underlying cause in arrhythmia management.

## Learning points

- Bidirectional ventricular tachycardia (BVT) has a narrow differential diagnosis; unexplained BVT accompanied by blood-pressure lability should prompt biochemical screening for pheochromocytoma.
- Beta-adrenergic blockade initiated before adequate alpha-blockade may unmask or precipitate malignant ventricular arrhythmias in unrecognized pheochromocytoma.
- Pheochromocytoma is a curable cause of malignant arrhythmia; tumor resection after appropriate alpha-blockade is definitive therapy, whereas antiarrhythmic drugs or device therapy alone are insufficient.

## Patient perspective

The patient initially sought medical attention for acute visual impairment caused by severely elevated blood pressure and was frightened when her heartbeat became irregular shortly after treatment began. The eventual diagnosis of pheochromocytoma provided clarity and relief. She expressed the hope that clinicians will consider reversible endocrine causes when confronted with unexplained malignant arrhythmias accompanied by blood-pressure fluctuations and reported that etiological diagnosis and curative surgery allayed her anxiety and restored her confidence in complete recovery. She has since returned to her usual daily activities and remains grateful that a reversible cause was found.

## Data Availability

The raw data supporting the conclusions of this article will be made available by the authors, without undue reservation.
